# High level of natural ionizing radiation at a thermal bath in Dehloran, Iran

**DOI:** 10.1016/j.heliyon.2020.e04297

**Published:** 2020-07-01

**Authors:** Mohammademad Adelikhah, Amin Shahrokhi, Stanislaw Chalupnik, Edit Tóth-Bodrogi, Tibor Kovács

**Affiliations:** a Institute of Radiochemistry and Radioecology, University of Pannonia, Egyetem u. 10, Veszprém, Hungary; b Silesian Centre for Environmental Radioactivity, Central Mining Institute, Plac Gwarków, 40-166, Katowice, Poland

**Keywords:** Radium concentration, Hot springs, Medical thermal bath, Radiation dose, Natural radon radiation, Environmental hazard, Environmental health, Environmental risk assessment, Natural hazard, Health sciences, Public health, Environmental science

## Abstract

It has been proven that more than half of the exposure to natural background radiation originates from radon isotopes and their decay products. The inhalation of radon and its decay products causes the irradiation of respiratory tracts, thus increasing the risk of lung cancer. In this study, the concentrations of radon and thoron in thermal baths at a spa in Dehloran (Iran) were investigated. The concentrations of dissolved ^226^Ra in samples of water from thermal baths were also measured. Additionally, the activity concentrations of abundant naturally occurring radionuclides in farmland soils irrigated with water from hot springs was measured and compared with other soil samples irrigated with water from other sources to estimate possible radioecological effects of natural radiation staff, patients and tourists at the spa are exposed to. In addition, the search for a link between the concentration of naturally occurring radionuclides in soil and the use of water from hot springs for irrigation was one of the main goals of the study. The activity concentrations of three major naturally occurring radionuclides in soil samples were measured; the ranges for ^40^K, ^226^Ra and ^228^Ra were 101 ± 8 to 240 ± 12, 276 ± 7 to 322 ± 12 and 20 ± 7 to 80 ± 10 Bq.kg^−1^, respectively. Higher activity concentrations of ^226^Ra and ^228^Ra were recorded in soil samples irrigated with hot spring water. The water from the same spring was used in all thermal baths so concentrations of dissolved ^226^Ra in water samples from different thermal baths were approximated to also be 0.42 ± 0.20 Bq.l^−1^. The indoor radon concentrations in the private thermal baths over a period of 45 days (including both occupied and vacant time) were measured to be between 1880 ± 410 and 2450 ± 530 Bq.m^−3^ and the radon concentrations in the spa galleries were measured to be between 790 ± 135 and 1050 ± 120 Bq.m^−3^, however, thoron concentrations were below the detection limit. The ventilation and centralized heating systems at the spa under investigation are inefficient so the radon concentrations in the therapy rooms and baths are high.

The maximum radiation doses originating from the inhalation of radon for tourists and the staff were estimated to be 0.13 and 5.5 mSv.yr^−1^, respectively, which is slightly over the national limit in Iran (5 mSv.yr^−1^). The exposure duration was estimated 15 and 1468 h per year for visitors and workers, respectively.

## 1 Introduction

Naturally occurring radiation can be defined as the radiation that originates from all sources in the natural environment including soil, water, the atmosphere or even space. The largest fraction of the annual effective dose the general population is exposed to from natural radioactivity originates from radon, thoron and their short half-life decay products. The decay products of radon are stated to be a major radiation hazard to humans, which increase the risk of lung cancer (EPA, 2003). For example, the annual effective dose of natural radiation that inhabitants in Ramsar (Iran) are exposed to is much higher than that of those in other parts of Iran, due to high concentrations of Naturally Occurring Radioactive Materials (NORM) in Ramsar (Harjo and Fervid, 2009). The hot spring and its surrounding area is an example of an elevated source of natural radiation that includes increased concentrations of ^222^Rn, ^226^Ra and ^228^Ra. The water from the hot spring is used extensively for balneotherapy and in thermal baths. These leisure activities are extremely popular in Europe and Asia as natural treatments for various common ailments. A large number of studies have been conducted on indoor radon concentrations in thermal spas, e.g. in the Middle-Slovakian region, Lądek-Zdrój in south-western Poland, the thermal spas of Rudas and Hévíz in Hungary, Jáchymov in the Czech Republic, etc (Szerbin et al., 1994; Lučivjanský; Ďurecová, 2000; Dombóváry et al., 2006; Jobbágy et al., 2010; Nowak et al., 2012; Zölzer et al., 2013; Walczak et al., 2016).

Nikolov et al. (2012) studied radon levels at the spa in Niška Banja, Serbia and measured activity concentrations indoors and in water in thermal pools and therapy rooms. The activity concentrations of NORMs in soil, rock and therapy mud were also recorded. Radon activity concentrations at spas of the Visegrad Group, namely in Poland, Hungary, the Czech Republic and Slovakia, have also been investigated using three types of passive radon detectors (RAMARN, RADUET and NRPB) (Műllerová et al., 2016a, Műllerová et al., 2016b).

In this paper, the activity concentrations of three of the most important naturally occurring radionuclides (^40^K, ^226^Ra and ^228^Ra) in soil samples from farmland near Dehloran which were irrigated by water from hot spring are presented. These soil samples were compared to those from the same area but irrigated by different sources of water. The results of the indoor ^222^Rn and ^220^Rn investigations in the thermal baths and therapy rooms at the spa are presented and the committed effective dose originating from the inhalation of decay products of radon estimated. Finally, the concentrations of ^226^Ra in aqueous form in water samples from the spa were measured.

Recommendations have been made by international organizations (WHO, ICRP) concerning the level of exposure to radon due to its high potential for increasing the risk of lung cancer. In December 2013, the Council of the European Union prepared the new European Basic Safety Standards (EU BSS). A permissible annual average radon concentration of no higher than 300 Bq.m^−3^ in dwellings and workplaces was established in this decree (EU BSS, 2014). Meanwhile, the International Atomic Energy Agency (IAEA), in accordance with the IAEA Safety Standard Series No. GSR Part 3, recommends that radon concentrations members of the general public and workers are exposed to do not exceed 300 Bq.m^−3^ and 1000 Bq.m^−3^, respectively (IAEA, 2014).

In Iran, the dose limit for workers, established in national regulations (ISIRI, 2005), is equal to 5 mSv.yr^−1^, therefore, it was important to estimate doses for the staff members of the spa as the most critical group. Furthermore, the doses that patients and tourists are exposed to has been estimated, taking into account the much shorter time of exposure, even for patients who regularly attend the baths.

## 2 Materials and methods

### 2.1 Study area

**Figure 1:**
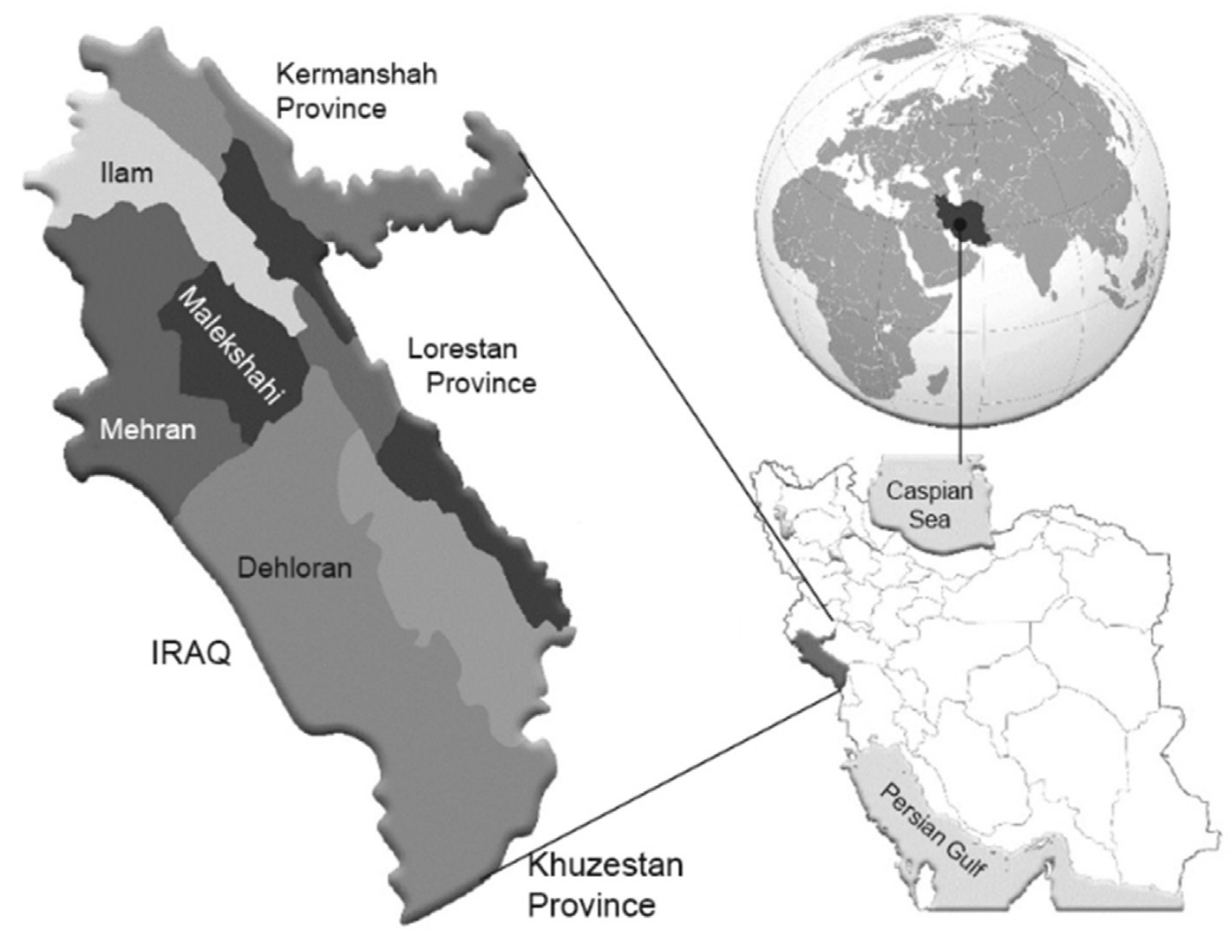
Study area.

Dehloran is a city in Ilam Province located in Western Iran (see Figure 1) and surrounded by mountains with a population of 66,000 inhabitants. The specific ecological environment of this area and its geology (rich in natural resources) are of particular importance to this city as they provide the opportunity for industrial development and tourism activities to stimulate and accelerate economic growth. Unique natural phenomena, e.g. natural tar spring, hot water springs and caves, deem this area ideal for research. Water from hot springs is used for medical treatments and irrigation. This particular spring is located 3 km from the center of Dehloran. The maximum temperature of the water in the spring is 50 °C. The study area is on the western part of the Zagros folded area. The age of the geological formations that outcropped in the region is related to the Cretaceous lower to the recent. From the old to the new, Sarvak, Pabdeh, Gurpi, Asmari, Gachsaran, Aghajari, and Bakhtiari formations occur in the study area (Mirzaee et al., 2019).

### 2.2 Sample preparation

Different locations inside the thermal bath in Dehloran were chosen for the measurements of indoor radon and thoron levels as well as the concentration of dissolved ^226^Ra in thermal water. The majority of these locations are where patients undergo treatments (baths as well as swimming pool areas). To determine the concentrations of radon and thoron, a RADUET selective radon and thoron chambers type with solid-state nuclear track detector CR-39 detector was used. The detectors were placed at least 40 cm away from doors and windows, 100–180 cm above the floor and about 8–15 cm away from any other objects for a period of 45 days. Water samples were preserved immediately after collection by 1N HNO_3_ to decrease the pH to 2 in order to avoid the absorption of radium into the walls of the bottles (EPA, 1980).

The soil samples from farms were also collected to determine the impact of thermal water used for irrigation on the distribution of naturally occurring radionuclides in the soil. All samples were randomly collected due to expected changes in the concentration of radionuclides with the distance from the source of water.

### 2.3 Gamma spectrometry

To measure spectra of gamma rays emitted from soil samples, 500 g of each sample was pulverized to less than 3 mm before being dried at 105 °C for 6 h to remove moisture. The samples were then placed into Marinelli beakers and sealed for approximately 29 days in order to reach secular equilibrium between ^226^Ra and ^222^Rn prior to counting (Schotzig and Debertin, 1983; Mollah et al., 1986; Maringer et al., 2013). The ^226^Ra activity concentration was determined via its decay products (295 keV of ^214^Pb and 609 keV of ^214^Bi), while the ^228^Ra activity concentration was measured from ^228^Ac gamma lines (911 keV and 969 keV). The activity concentration of ^40^K was measured from its 1460 keV direct gamma line. A semiconductor High Purity Germanium (HPGe) detector took the measurements (ORTEC GMX40-76, efficiency of 40%). The data and spectra were recorded by an ORTEC DSPEC LF 8196 MCA. The system was calibrated with an IAEA-375 soil reference material with known activity concentration of radionuclides (IAEA, 2007).

### 2.4 ^226^Ra content in water samples

The concentration of ^226^Ra in water samples was measured using the emanation method with the application of a RAD7, an electronic radon detector with H_2_O accessories (DURRIDGE). In this method, 2–4 L of water was slowly reduced to 300 mL by evaporation. The sample was bubbled for about 10 min using a radon-free gas like nitrogen to eliminate all the radon in the water. Then, the sample was sealed for about 29 days to reach secular equilibrium between the parent nuclide ^226^Ra and its decay products. The RAD H_2_O accessory employs a closed loop aeration scheme whereby the volumes of air and water are constant and independent of the flow rate (Nikolov et al., 2012). The activity concentration of radon in the water was calculated using CAPTURE RAD7 data Acquisition and Analysis Software. The calculations were based on Eq. (1) (Bill et al., 1998; DURRIDGE, 2013):

(1) C_w_ = [C_a_ (V_a_ + KV_w_) − C_b_V_a_] / (V_w_ − V_h_/K)

where C_w_ denotes the radon concentration in water (Bq.l^−1^), C_a_ represents the activity concentration of radon in air, C_b_ stands for the background radiation, V_a_, V_h,_ and V_w_ are the total volumes of the system, headspace in the bottle and sample (L), respectively, and K denotes the division factor of radon between the aqueous and gaseous phases calculated from the Fritz von Weigel relation as 0.105 + 0.405e^(−0.0502T)^ where T represents the water temperature (ºC).

The concentration of ^226^Ra in the water was assumed to be the same as the concentration of radon ^222^Rn.

### 2.5 ^222^Rn content in water samples

The concentration of ^222^Rn in water samples was measured by the emanation method with the application of RAD7 using a similar method to measurements of ^226^Ra in water. Samples of water were poured into 300-milliliter containers before the air was circulated in the device to allow the bubbling action to release the radon from the water and the concentration of radon to be measured according to Eq. (1).

A number of factors affect the accuracy and precision of radon in water measurements. One of the most critical of which is the sampling technique but others include the sample size, counting time, temperature, relative humidity and background effects (Nikolov et al., 2012). For the measurements, the samples were collected according to the technique proposed by the manufacturer (DURRIDGE, 2013).

### 2.6 Indoor radon and thoron concentrations

To determine the indoor radon and thoron concentrations, passive radon-thoron discriminative detectors, commercially known as RADUET (Radosys Ltd.), were used. Such detectors were used to measure not only the concentrations of radon but thoron as well. These detectors consist of two diffusion chambers with different ventilation rates, each of which contains a CR-39 chip, the dimensions of which are 10 × 10 mm, to detect alpha particles emitted by radon and thoron as well as their decay products (Tokonami et al., 2005; Shahrokhi et al., 2016). The low diffusion rate chamber is comprised of an electrically conductive plastic with an inner volume of 30 cm^3^. Six holes in the wall of the high diffusion rate chamber, composed of the same material, are covered with a sponge material to prevent decay products of radon and thoron as well as aerosols from infiltrating into the chamber. While one chamber only measures radon, the other detects both radon and thoron levels. The concentration of thoron is then determined by subtracting the track densities. After exposure, all detectors were wrapped in protective aluminum foil and returned for processing at the Institute of Radiochemistry and Radioecology at the University of Pannonia.

In the laboratory, detectors were chemically etched in a 6 M NaOH solution for 3 h at 90 °C before being washed with distilled water and dried. The track densities were counted using a high-resolution image scanner and image analysis software (Bátor et al., 2015). The concentration of radon was calculated using Eq. (2):

(2) C_Rn_ = (N_t_ − N_b_) × E/(T × A)

where C_Rn_ denotes the average indoor radon concentration (Bq.m^−3^), N_t_ stands for the total number of tracks, N_b_ represents the number of background tracks, E is the calibration factor (Bq.m^−3^.h.tracks^−1^.mm^2^), T denotes the exposure period (hours), and A stands for the reading area of the tracks (mm^2^).

In order to determine the calibration factor of track detectors, several CR-39 detectors were placed in a Certified Radon Chamber (CRC) with an AlphaGUARD PQ2000PRO radon monitor as a reference instrument. Detectors were exposed to a stable ^222^Rn concentration of 2,000 Bq.m^−3^ for 6 days. To create a stable atmosphere of radon inside the chamber, a ^226^Ra standard source (104.9 kBq ±0.4 %), manufactured by Pylon Electronics Inc., was used.

### 2.7 Estimation of the radiation dose caused by radon

The annual effective dose for people in spas was calculated according to parameters proposed by the United Nations Scientific Committee on the Effects of Atomic Radiation (UNSCEAR) (UNSCEAR, 2008, 2010):

(3) H (mSv.yr^−1^) = C_Rn_ × F × O × (DCF)

where C_Rn_ denotes the average indoor radon concentration (Bq.m^−3^), F represents the equilibrium factor between radon and its decay products (0.4), O stands for the average indoor occupancy time per person (for visitor, the exposure duration was estimated 1 time a year for 3 days and spending each day about 5 h in bath = 15 h per yearly visit; in case of workers based on Iranian working hour, estimated as 1835 hourd per year by assuming 80% of their working time spend in the bath area) and DCF is the dose conversion factor for radon exposure which was taken from Iranian regulation as 9×10^−6^ mSv.h^−1^.Bq^−1^.m^3^ based on UNSCEAR, therefore, the new ICRP value is not applied yet in Iranian regulation. However, to compare the difference between new ICRP DCF (6.7×10^−6^ mSv.h^−1^.Bq^−1^.m^3^) and Iranian DCF taken from UNSCEAR, doses were calculated based on these two DCFs and are given in Table 2 (UNSCEAR, 2017; ICRP, 2017).

## 3 Results and discussion

**Figure 2:**
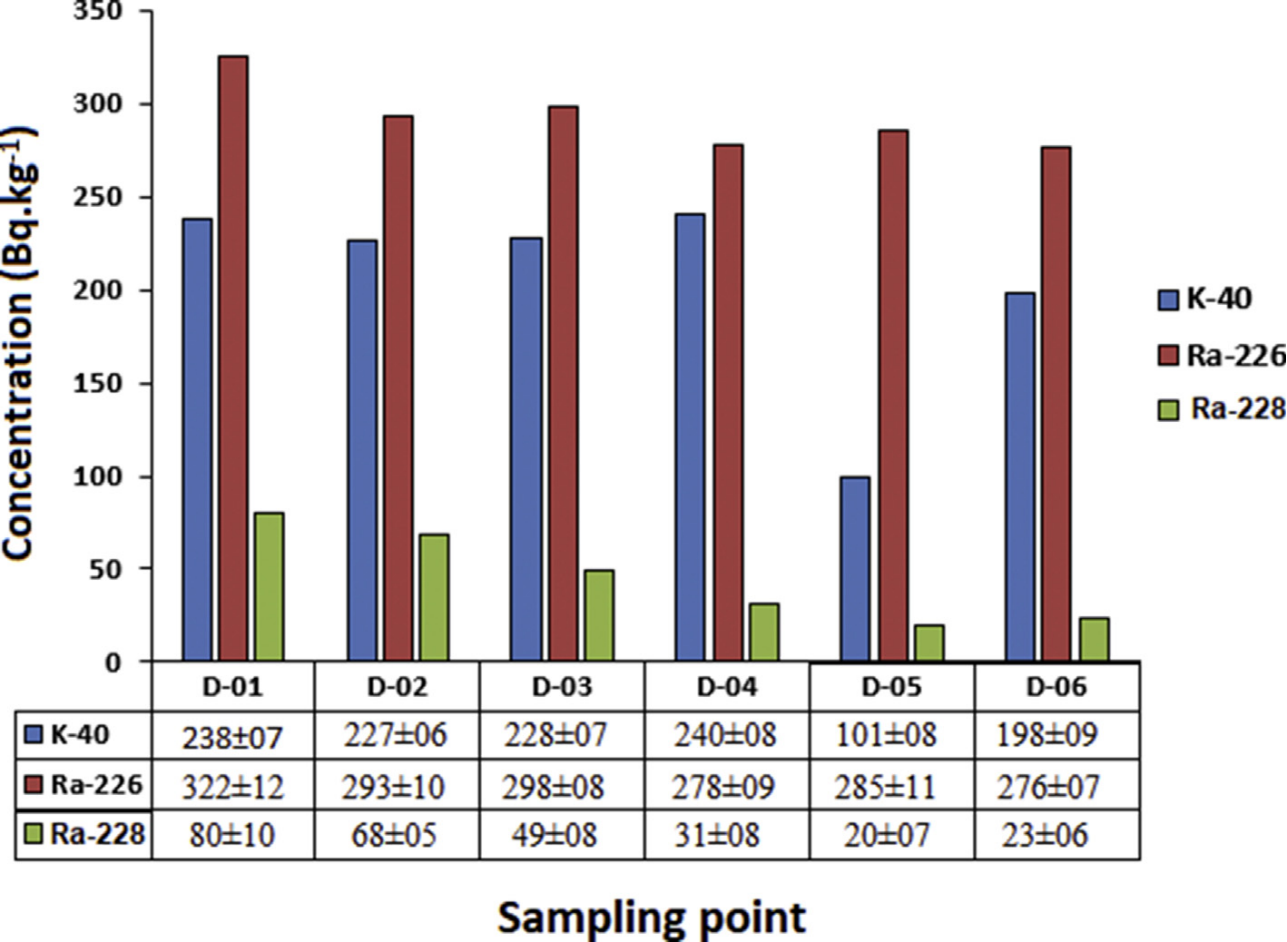
Concentration of ^40^K, ^226^Ra and ^228^Ra in soil samples irrigated by different sources of water.

In Figure 2, the concentrations of ^40^K, ^226^Ra and ^228^Ra present in the soil samples are shown. The Minimum Detectable Concentrations (MDC) for particular radionuclides were calculated as 46 Bq.kg^−1^ for ^40^K, 1.3 Bq.kg^−1^ for ^226^Ra and 2.3 Bq.kg^−1^ for ^228^Ra. Soil samples D-01 to D-03 were collected from different areas of land where hot spring water was used for irrigation, while soil samples D-04 to D-06 were extracted from reference areas where another source of water was used. The results shown in Figure 2 reveal that the average concentration of ^40^K is below the worldwide average, whereas the mean concentrations of ^228^Ra (^232^Th) and ^226^Ra are much higher than it. Values of 400, 32 and 30 Bq.kg^−1^ are the world average concentrations of ^40^K, ^226^Ra and ^228^Ra in soil, respectively (UNSCEAR, 2008). For example, the mean concentration of ^226^Ra in the soil samples is 8 times higher than the world average. The results show that a systematic relationship between the concentrations of ^226^Ra and ^228^Ra in the soil samples and sources of hot water. In fact, the soil samples which were collected from other areas of farming land supplied by different sources of water for irrigation close to the sampling area of this study consist of lower concentrations of ^226^Ra and ^228^Ra. Probably the reason for this was the presence of ^228^Ra in the water (unfortunately, this was impossible to measure using gamma spectroscopy due to its detection limit). This is also why the concentration of ^228^Ra but not ^232^Th was presented, due to possible disequilibrium of the thorium series.

**Figure 3:**
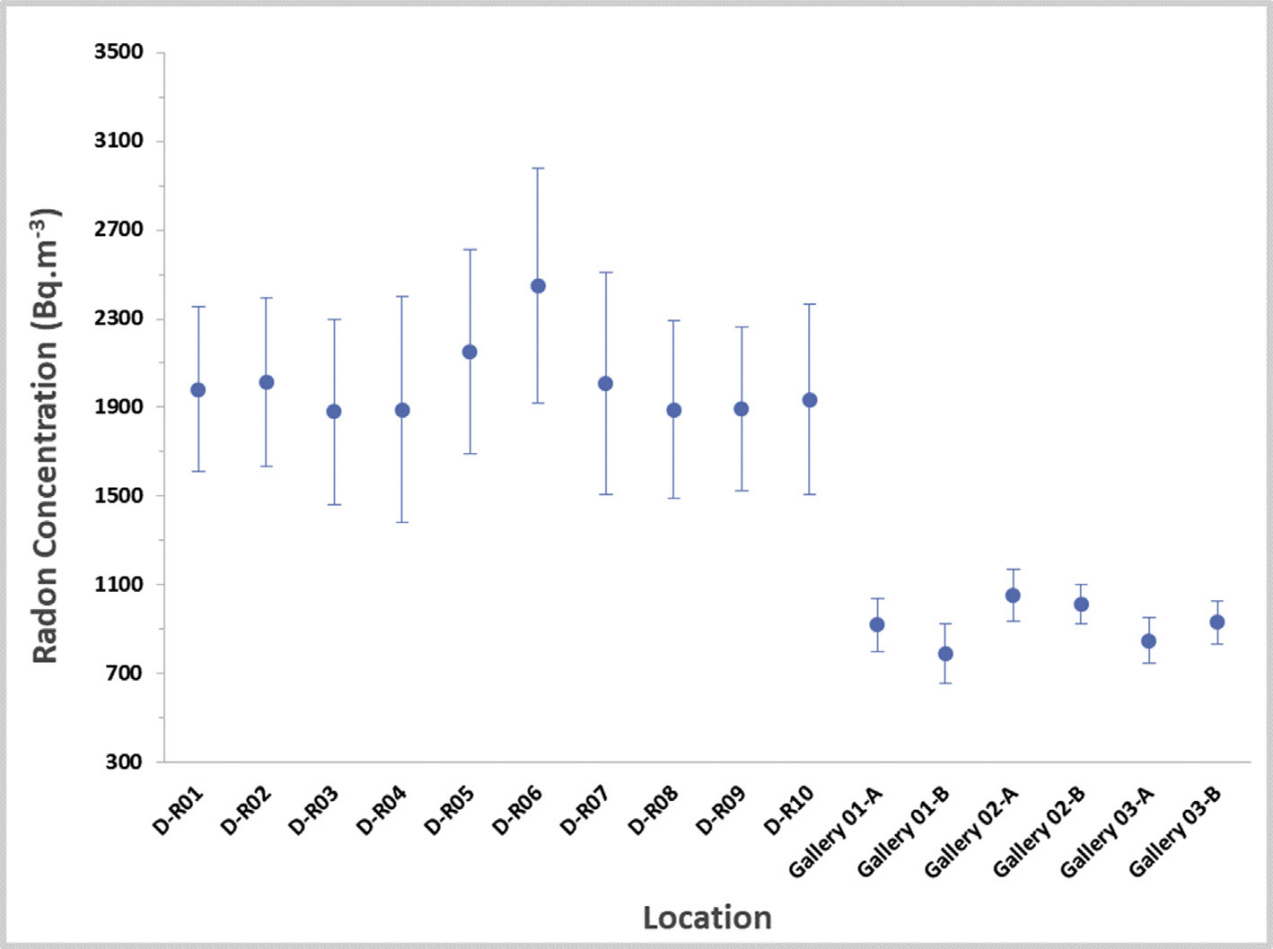
Activity concentrations of ^222^Rn in thermal baths.

Visitors and workers who use the baths served by the hot springs are also exposed to radiation through the inhalation of radon. During showering, water splashes and radon is released into the air resulting in possible inhalation exposure. In addition, the use of building materials and decorative stones is another source of indoor exposure. Figure 3 shows the concentration of ^222^Rn for each bath and gallery. The concentrations of ^222^Rn in the baths measured were between 1880 ± 410 and 2450 ± 530 Bq.m^−3^ with an average of 2010 ± 440 Bq.m^−3^. Unfortunately, due to high radon concentrations, all the thoron concentration results were below the detection limit of the method. It is interesting to note that the radon concentrations in the galleries were less than those in the baths, ranging from 790 ± 135 to 1050 ± 120 Bq.m^−3^, as the ventilation system causes a reduction in the radon concentration in air. In addition, each of the baths in this thermal spa only has one small window, therefore, are characterized by high relative humidity due to the inability of the poor ventilation system to efficiently refresh the air inside the premises. In this thermal bath, no storage reservoirs for water are present, unlike in other modern spas. The ^222^Rn concentrations are significantly higher when compared with those observed in modern spas elsewhere (see Table 1: Sohrabi, 1997; Radolić et al., 2005; Song et al., 2005; Beitollahi et al., 2007; Ródenas et al., 2008; Bituh et al., 2009; Onishchenko et al., 2010; Nikolov et al., 2012; Pugliese et al., 2013; Kasić et al., 2015; Műllerová et al., 2016; Uzun and Demiröz, 2016; Karakaya et al., 2017; Silva and Dinis, 2017; Wang et al., 2017).

**Table 1:** The concentration of radionuclides in geothermal water or hot springs in different countries.

Study Area	^226^Ra in water (mBq.l^−1^)	^222^Rn in water (Bq.l^−1^)	Indoor radon (Bq.m^−3^)	Reference
**This study**	408–423	83–168	1880–2450	Current study
Bosnia and Herzegovina	1–791	---	---	(Kasić et al., 2015)
china	23–363	0.47–29.70	40–2855	(Wang et al., 2017; Song et al., 2005)
Czech Republic	---	50->300	120–3100	(Műllerová et al., 2016)
Croatia	87–6200	---	10.9–109	(Bituh et al., 2009; Radolić et al., 2005)
France	278–865	1.7–10.9	---	(Condomines et al., 2012)
Hungary	321–1099	1–100	<20-540	(Műllerová et al., 2016)
Italy	---	7–98	30–3983	(Pugliese et al., 2013)
Iran (Mahallat)	480–1350	145–2731	275–700	(Beitollahi et al., 2007)
Iran (Ramsar)	1000–146000	1–160	---	(Sohrabi, 1997)
Poland	13–808	0–20	<25-100	(Műllerová et al., 2016)
Portugal	---	26–6949	73–4335	(Silva et al., 2016; Silva and Dinis, 2017)
Romania	920–1100	20.15–26.94	---	(Roba et al., 2010)
Russia	50–4100	---	---	(Onishchenko et al., 2010)
Serbia	---	24.5–648	140–2810	(Nikolov et al., 2012)
Slovakia	---	4.1–488	90–12100	(Műllerová et al., 2016; Blahušiak et al., 2017)
Spain	4–3660	<4-1868	---	(Ródenas et al., 2008)
Turkey	1385–11025	0.21–5.82	13–10000	(Karakaya et al., 2017; Uzun and Demiröz, 2016; Tabar and Yakut, 2014)

Results from the measurements of the activity concentrations of ^222^Rn in water samples are presented in Table 1. It can be seen that the activity concentrations of radon in the water samples were clearly elevated, reaching 168 ± 18 Bq.l^−1^. As a result, water is the main source of radon in the baths amongst other places. Furthermore, a reduction in the radon level in water as its distance from the hot spring increases was visible which explains why the radon concentration decreased in baths located further from the source.

It is necessary to mention that due to time and budget limitations, measurements of the radon level in other locations in this area were omitted. However, it was found that enhanced radon concentrations were generated by ^222^Rn dissolved in hot spring water and released in baths amongst other places. Secondly, the thermal water does not constantly run into baths but its flow depends on the usage of baths by clients. Results have shown correlations between the concentration of ^222^Rn and the size of baths, the frequency of their use and their distance from a hot spring. It is evident that the concentration of radon in the indoor air of the Spa in Dehloran varied from one treatment room to another, due to the influence of several factors, e.g. its size, the ventilation rate and water flow rate. The highest concentration of 2450 ± 530 Bq.m^−3^ was measured in one of the baths.

The maximum radiation dose for the members of staff at the spa as a result of inhaling radon was estimated based on the measured results and recommended maximum treatment duration (see Table 2). If the percentage of the spending time of staff in the bath area is 80%, the actual dose of radon received by staff was estimated to be within the range of 4.16–5.55 mSv.yr^−1^ with an average of 4.9 mSv.yr^−1^. The minimum and maximum annual radiation doses originating from radon received by tourists was estimated to be 0.043–0.132 mSv.yr^−1^, respectively, with an average of 0.086 mSv.yr^−1^. It was also estimated that for the most frequent clients of the spa, the annual radiation dose should not exceed 10 % of the dose the staff are exposed to, i.e. 0.55 mSv.yr^−1^.

**Table 2:** The annual dose caused by radon with regard to tourists and staff at the spa.

Location	C_Radon_ (Bq.m^−3^)	C_Thoron_ (Bq.m^−3^)	Dose (mSv.yr^−1^) (UNCEAR DCF)		Dose (mSv.yr^−1^) (ICRP 137 DCF)	
			Tourist	Staff	Tourist	Staff
D-R01	1982 ± 370	<209	0.107	-	0.079	-
D-R02	2012 ± 380	<211	0.108	-	0.080	-
D-R03	1880 ± 420	<204	0.101	-	0.075	-
D-R04	1890 ± 510	<205	0.102	-	0.075	-
D-R05	2150 ± 460	<218	0.116	-	0.086	-
D-R06	2450 ± 530	<232	0.132	-	0.098	-
D-R07	2010 ± 500	<211	0.108	-	0.080	-
D-R08	1890 ± 400	<205	0.102	-	0.075	-
D-R09	1895 ± 370	<205	0.102	-	0.076	-
D-R10	1937 ± 430	<207	0.104	-	0.077	-
Gallery 01-A	920 ± 120	<143	0.049	4.86	0.036	3.62
Gallery 01-B	789 ± 135	<133	0.043	4.16	0.031	3.1
Gallery 02-A	1052 ± 120	<153	0.056	5.55	0.042	4.13
Gallery 02-B	1014 ± 90	<151	0.054	5.35	0.040	3.99
Gallery 03-A	850 ± 100	<138	0.045	4.49	0.034	3.34
Gallery 03-B	932 ± 98	<144	0.050	4.92	0.037	3.66

The measured concentrations of ^226^Ra in water samples are shown in Table 3. The concentrations of ^226^Ra in the samples of thermal water were measured within the range of 408 ± 200 to 423 ± 180 mBq.l^−1^ with an average of 419 ± 170 mBq.l^−1^. Since the water source is the same for the baths and thermal pools, the concentration of ^226^Ra fell approximately within the same range. If these values are compared with the results shown in Table 1 in which the reported indoor radon and ^226^Ra activity concentrations in water in various spas around the world are presented, it can be seen that the concentration of this radionuclide is elevated compared to typical levels in surface waters (Radolić et al., 2005; Song et al., 2005; Beitollahi et al., 2007; Ródenas et al., 2008; Bituh et al., 2009; Onishchenko et al., 2010; Nikolov et al., 2012; Pugliese et al., 2013; Kasić et al., 2015; Műllerová et al., 2016; Uzun and Demiröz, 2016; Karakaya et al., 2017; Silva and Dinis, 2017; Wang et al., 2017) but similar to radium levels in other spas.

**Table 3:** ^226^Ra concentrations in water samples.

Location	^226^Ra (mBq.l^−1^)	SD
D-R01	418	±180
D-R02	421	±170
D-R03	423	±160
D-R04	421	±165
D-R05	422	±180
D-R06	428	±188
D-R07	408	±200
D-R08	415	±145
D-R09	413	±130
D-R10	416	±183
Gallery 01	422	±190
Gallery 02	419	±170
Gallery 03	423	±180
**Mean**	**419.15**	**±171**

## 4 Conclusions

The concentration of radon in baths and galleries at the spa in Dehloran fell within the range of 1880–2450 Bq.m^−3^ and 790 ± 135 to 1050 ± 120 Bq.m^−3^, respectively, which is significantly higher than the recommended values.

Measurements of ^222^Rn concentration in water samples were taken. The maximum concentration of radon in water was 168 ± 18 Bq.l^−1^, moreover, a reduction in the radon concentration in water as the distance from its source increased was observed.

The results of thoron concentration measurements in baths fell below the detection limit due to high ^222^Rn concentrations making discriminative measurements of ^220^Rn impossible. This is a very important result that illustrates how crucial lower limits of detection (LLD) of thoron in the presence of high concentrations of ^222^Rn are.

The maximum radiation dose of the staff can be exposed to as a result of inhaling radon was estimated based on measurements and the estimated maximum working time indoors. The annual radiation dose caused by radon in the case of the staff was on average 4.89 mSv.yr^−1^, with a maximum value of 5.55 mSv.yr^−1^. This shows the importance of determining the dose workers are exposed to and for whom the radiation hazard of radon is more significant than for tourists (0.55 mSv.yr^−1^). The annual radiation dose that originates from radon for tourists was also estimated to be between 0.043 and 0.132 mSv.yr^−1^ with an average of 0.086 mSv.yr^−1^.

The activity concentrations of abundant naturally occurring radionuclides in soil samples from the vicinity of the thermal spa in Dehloran were measured. It was found that the activity concentrations of these radionuclides in locations where water from the hot spring or other sources was used for irrigation were different. In these samples, the concentrations measured fell within the range of 227–238 Bq.kg^−1^ for ^40^K, 293–322 Bq.kg^−1^ for ^226^Ra and 49–80 Bq.kg^−1^ for ^228^Ra. Lower values were measured in soil samples from land irrigated by water from other sources, 101–240 Bq.kg^−1^, 276–285 Bq.kg^−1^ and 20–31 Bq.kg^−1^ for ^40^K, ^226^Ra and ^228^Ra, respectively, to be exact. This is indicative of a direct correlation between the activity concentrations of ^226^Ra and ^228^Ra in the soil and water source used for irrigation even though the concentrations of ^228^Ra were not measured.

Therefore, it is necessary to mitigate this dose by increasing ventilation and/or aerating the spring water to remove radon. Since the maximum estimated dose for members of staff was close to the national dose limit, environmental radon monitoring at the spa must be implemented with the provision of personal dosimetry for the members of staff most at risk. Given the results of this study, the implementation of a regulation for monitoring radon concentrations is highly recommended in popular public places such as thermal baths and caves.

## Declarations

### Author contribution statement

Mohammademad Adelikhah, Amin Shahrokhi: Conceived and designed the experiments; Performed the experiments.

Stanislaw Chalupnik: Analyzed and interpreted the data; Wrote the paper.

Edit Tóth-Bodrogi: Analyzed and interpreted the data; Contributed reagents, materials, analysis tools or data; Wrote the paper.

Tibor Kovács: Conceived and designed the experiments; Analyzed and interpreted the data; Contributed reagents, materials, analysis tools or data; Wrote the paper.

### Funding statement

This work was supported by Hungarian National Research OTKA grant No. K128805, K128818 and GINOP Grant of the 10.13039/501100015269Hungarian Government No. 2016-0016.

### Competing interest statement

The authors declare no conflict of interest.

### Additional information

No additional information is available for this paper.
